# Exploring the Nutritional and Bioactive Potential of Olive Leaf Residues: A Focus on Minerals and Polyphenols in the Context of Spain’s Olive Oil Production

**DOI:** 10.3390/foods13071036

**Published:** 2024-03-28

**Authors:** Carolina L. Ronca, Carmen Duque-Soto, Cristina Samaniego-Sánchez, María Encarnación Morales-Hernández, Manuel Olalla-Herrera, Jesús Lozano-Sánchez, Rafael Giménez Martínez

**Affiliations:** 1 Department of Pharmacy, University of Federico II of Naples, 80138 Naples, Italy; carolinaronca@correo.ugr.es; 2 Department of Nutrition and Bromatology, Faculty of Pharmacy, University of Granada, 18012 Granada, Spain; carmenduque@ugr.es (C.D.-S.); csama@ugr.es (C.S.-S.); olalla@ugr.es (M.O.-H.); rafaelg@ugr.es (R.G.M.); 3 Department of Pharmaceutical Technology, Faculty of Pharmacy, University of Granada, 18012 Granada, Spain; maen@ugr.es

**Keywords:** olive phenols, oleuropein, olive leaf extract, green extraction, ionic gelation, microencapsulation, functional yogurt, goat yogurt

## Abstract

Lyophilized plant-origin extracts are rich in highly potent antioxidant polyphenols. In order to incorporate them into food products, it is necessary to protect these phytochemicals from atmospheric factors such as heat, light, moisture, or pH, and to enhance their bioavailability due to their low solubility. To address these challenges, recent studies have focused on the development of encapsulation techniques for antioxidant compounds within polymeric structures. In this study, lyophilized olive leaf extracts were microencapsulated with the aim of overcoming the aforementioned challenges. The method used for the preparation of the studied microparticles involves external ionic gelation carried out within a water–oil (W/O) emulsion at room temperature. HPLC analysis demonstrates a high content of polyphenols, with 90% of the bioactive compounds encapsulated. Meanwhile, quantification by inductively coupled plasma optical emission spectroscopy (ICP-OES) reveals that the dried leaves, lyophilized extract, and microencapsulated form contain satisfactory levels of macro- and micro-minerals (calcium, potassium, sodium). The microencapsulation technique could be a novel strategy to harness the polyphenols and minerals of olive leaves, thus enriching food products and leveraging the antioxidant properties of the polyphenolic compounds found in the lyophilized extract.

## 1. Introduction

Olive trees are one of the main crops in the European countries of the Mediterranean basin. In this sense, olive oil production has held significant influence over the history, economy, and culture of Western civilization [1]. The association of its consumption with numerous positive effects on human health linked with the benefits of the Mediterranean diet has led to its increased consumption. Among the Mediterranean countries, Spanish production involves a high percentage of the olive oil produced worldwide. In this sense, its olive cultivation covers an area of 2.75 million hectares, which accounts for 70% of EU production and 45% of global production [2,3].

As a consequence of the increased demand and production, vast quantities of derived residues are obtained. These diverse residues range from those obtained in the agricultural phase, such as leaves and branches, to those derived from the olive oil production process, including olive pomace and oil mill wastewater [4]. Indeed, in the EU-28, olive mills and tree-pruning-derived residues account for more than 20 million tons/year [5]. Their management by olive producers has proved to be both an economic and environmental challenge as their toxicity related to their high content in phenolic compounds and organic matter establishes their traditional disposal as accumulated in the field or burning as potential pollutant sources. In this sense, their accumulation has proved to be a pressing environmental issue related to their high organic composition, as they have been associated with aquifer contamination, eutrophication, and odor problems [6]. Olive leaves, an abundant waste product of tree pruning, stand out as one of the primary contributors to this waste generation. Indeed, an annual production of 25 kg of waste, including leaves and branches, per olive tree has been estimated [7]. The conventional method for their removal has entailed the grinding and incineration of these by-products, with a derived severe environmental impact. Thus, the optimal management of these wastes has gained interest in the scientific community, with a great interest in the development of a circular economy.

In this sense, olive leaves are presented as an unexploited and novel source of bioactive compounds, with both interesting technological and health properties, as they have been traditionally used for medicinal applications in folk medicine [8]. Among the bioactive composition, phenolic compounds, a heterogeneous group of molecules derived from plants’ secondary metabolism elated to their response against both biotic and abiotic stressors, have garnered significant scientific interest over the last decade for their bioactive status. These compounds are abundantly present in olive leaves, including phenolic alcohols, phenolic acids, flavonoids, and secoiridoids [9,10]. 

The latest phenolic -family has only been identified in the Oleaceae family and is abundantly present in the evaluated by-product. Specifically, oleuropein being the most abundant phenolic compounds in the olive leaf, and its precursor hydroxytyrosol, have presented great bioactive potential. Indeed, olive leaf extracts have been related to a variety of biological activities in relation to their mentioned phenolic content, such as antioxidant, anti-inflammatory, anti-microbial, and antiviral, as well as anti-atherogenic, anti-cancer, hypolipidemic, and hypoglycemic effects and have had an impact on several intestinal pathologies as related to a modulatory effect on intestinal microbiota due to their prebiotic effect [11,12,13,14,15]. Additionally, extracts from these by-products have also been applied in the cosmetic and food industries, as additives to increase food shelf-life and safety [16,17,18]. Therefore, their revalorization as a potential source of bioactive molecules could lead to the obtention of high added-value products, boosting the industry, and reducing the environmental impact derived from their accumulation.

Given their bioactive properties, one of the main areas of potential applications is in their incorporation into functional foods, in which phenolic compounds could serve both health and technological purposes, such as color retention and delaying microbial growth and lipid oxidation [19,20,21,22]. Thus, phenolic extracts have been included in multiple food products, from refined oils to dressings and fruit smoothies [23]. In this sense, fermented products such as dairy products have surged as some of the most interesting incorporating matrices due to the potential interaction of these bioactive compounds with the inherently present probiotic strains. However, although highly interesting, incorporation of olive leaf extracts into raw materials for the production of functional foods is underexplored, as phenolic compounds are highly unstable under environmental and processing conditions, being sensitive to both light, pH, and high temperatures [24]. 

Encapsulation techniques have been developed as alternative delivery strategies allowing for the protection of phenolic compounds during processing, before their introduction into a food matrix [25,26]. This delivery system can allow for the incorporation of bioactive compounds without altering their attributes, their protection from external conditions, and a controlled release which may positively influence the food matrix in which it is introduced. In this sense, alginate-based ionic gelation encapsulation systems present an incredible potential as this polysaccharide has been widely used in the food industry and exhibits simplicity. Sodium alginate is a polymer widely used as a microencapsulating agent, since it has a high affinity for water, forming a homogeneous, non-toxic, and biodegradable gel [27]. In addition, it provides a prebiotic effect if ingested and also acts as dietary fiber, reducing blood glucose and cholesterol [28].

Thus, the main aim of this study was the revalorization of olive leaves by-product through: (a) the obtention of a phenolic-rich extract; (b) stabilization of bioactive compounds by ionic gelation encapsulation; and (c) incorporation of microencapsulated olive leaves phenolic-rich extract into dairy fermented product. To achieve this goal, solid–liquid extraction and encapsulation through alginate-in-oil emulsion coupled with ionic gelation were proposed for developing functional ingredients. Validation of the microencapsulated formulation was carried out via incorporation into the goat yogurt production process with the intention of evaluating the potential of this encapsulation strategy on the development of a functionalized food product. Additionally, the effect of the inclusion of the obtained microparticles into the micronutrient content of the functional yogurt was evaluated. 

## 2. Materials and Methods

### 2.1. Chemicals and Reagents

All reagents and solvents were of analytical or HPLC grade. For phenolic extraction, ultrapure water was obtained with a Milli-Q system (Millipore, Bedford, MA, USA), and absolute ethanol (EtOH) and formic acid were purchased from Panreac (Barcelona, Spain). As for HPLC analysis, methanol (MeOH) was obtained from Honeywell (Seelze, Germany). Standards of oleuropein, rutin, hydroxytyrosol, gallic acid, p-coumaric acid, and diosmin were obtained from Sigma-Aldrich (St. Louis, MO, USA); ferulic acid, chlorogenic acid were purchased from Fluka Chemie GmBH (Buchs, Switzerland), and verbascoside was acquired from PhytoLab GmbH & Co. KG (Vestenbergsgreuth, Germany).

For microparticles preparation, the following reagents were provided by the mentioned suppliers: glacial acetic acid (VWR Chemicals BDH, Milan, Italy), sodium alginate (Fagron Ibérica S. A., Barcelona, Spain), soya oil (GUINAMA, Valencia, Spain), SPAN 80, calcium chloride (CaCl_2_) and calcium Carbonate (CaCO_3_) (Sigma-Aldrich, St. Louis, MO, USA). About the preparation of the yogurt, probiotic yogurt cultures YO-MIX 205 LYO 250 DCU were obtained from Danisco (Dange-Saint-Romain, France).

Concerning antioxidant capacity assays, trolox (6-hydroxy-2,5,7,8-tetramethylchroman-2-carboxylic acid), ABTS [2,2′-azinobis(3-ethylbenzothiazoline-6-sulphonate)], DPPH (2,2-Diphenyl-1-picrylhydrazyl), and TPTZ (2,4,6-tripyridyl-S-triazine) were purchased from Sigma-Aldrich (St. Louis, MO, USA); potassium persulphate and copper (II) sulphate 5-hydrate were purchased from Panreac (Barcelona, Spain).

For mineral analysis, certified single-element standards provided by Perkin-Elmer (Perkin Elmer, Inc., Waltham, MA, USA) were used for calibration line, and nitric acid was provided from Honeywell (Seelze, Germany).

### 2.2. Plant Material

Fresh olive leaves (*Olea europaea* Sativa Picual variety) were collected in the region of Granada (Spain) in March of 2022. The olive leaves (OL) were dried at room temperature for 2–3 days, away from sources of light or heat and in a cool, non-humid place, to remove water and concentrate the compounds present inside the leaves. Then, prior to the extraction, dried olive leaves (DOL) were ground to obtain leaf powder using a conventional coffee mill. Furthermore, leaves were stored for a 2-month period for analysis of phenolic compounds’ evolution.

### 2.3. Olive Leaf Solid–Liquid Extraction

Macerations were carried out in triplicate using the optimal solvent conditions as described in Ronca et al., 2024 [29]. Briefly, different proportions of olive leaf powder (2, 5, 12.5 g) were mixed with 100 mL of EtOH/H_2_O 1:1 with 0.1% of formic acid. The solutions were shaken in a vortex for 2 min and introduced in an ultrasound bath for 30 min. Afterwards, the mixtures were mechanically shaken for 40 min and centrifuged at 5000 rpm, 20 °C, and 30 min. The supernatants were lyophilized in a Zirbus freeze-dryer (Zirbus Vaco 2, Bad Grund, Germany) for 96 h and stored for later analyses (antioxidant capacity and HPLC).

### 2.4. In Vitro Antioxidant Activity

The antioxidant capacity was evaluated through FRAP (Ferric Reducing Antioxidant Capacity), DPPH (2,2-diphenyl-1-picrylhydrazyl radical), and TEAC (Trolox Equivalent Antioxidant Capacity) assays. Analyses were carried out as triplicates in a microplate reader from Synergy MX, BioTek (Winooski, VT, USA).

FRAP assay was performed as described by Benzie and Strain et al., 1996, with slight modifications [30]. Briefly, FRAP reagent was prepared by mixing acetate buffer pH 3.6, TPTZ 10 mM in HCl 40 mM, and 20 mM of FeCl_3_-6-H2O (10:1:1, *v*/*v*/*v*). Extract dilutions (200 µL) were added to 1.5 mL of radical FRAP reagent and incubated for 10 min at room temperature. Solutions absorbance was measured at 593 nm. Antioxidant capacity was determined by interpolating in the calibration curve obtained using different Trolox concentrations and presented in Supplementary Materials (Table S1) (10, 20, 25, 30, 50, 70, 100 mM) (y = 6.1115x − 0.0235; R^2^ = 0.999). FRAP values were expressed as µmol FeSO_4_ equivalents/g lyophilized extract. 

DPPH assay was performed using the procedure described by [30]. Briefly, 50 µL of extract (3 mg in 1 mL of EtOH/H_2_O 50:50) was added to 1.45 mL of DPPH solution (2.3 mg in 100 mL of MeOH). Solutions were incubated for 15 min at room temperature, and the absorbance was measured at 515 nm. A calibration curve was obtained using different Trolox concentrations (50, 100, 250, 500 mM) (y = 146.71x + 2.5309; R^2^ = 0.997), presented in Supplementary Materials (Table S1). The results were expressed as µmol Trolox equivalent per gram (µmol TE/g). 

TEAC (Trolox Equivalent Antioxidant Capacity) assay was developed as previously reported [8]. The ABTS reagent was prepared by mixing 5 mL of 7 mM ABTS radical with 88 µL of 140 mM potassium persulphate K_2_S_2_O_8_ (1:0.5, *v*/*v*). The mixture was kept in the dark for 12–16 h for maximum stability and absorbance of the radical. Once the radical was formed, it was diluted until its absorbance was 0.700 ± 2 at 734 nm. Appropriate sample dilutions (200 µL) were added to 2 mL of FRAP of ABTS·+. The absorbance was measured at 734 nm once per min for 30 min at 25 °C and compared to a Trolox calibration curve (0.01, 0.1, 0.2, 0.3, 0.4 mM) (y = 237.19x + 1.0381; R^2^ = 0.9975), presented in Supplementary Materials (Table S1). The results were expressed as µmol Trolox equivalents/g of lyophilized extract.

### 2.5. Microencapsulation of Olive Leaf Extracts by Ionic Gelation

For preparation of the aqueous phase for microparticles preparation on a water–oil (W/O) emulsion, the lyophilized extract (50 mg), sodium alginate (1 g), and calcium carbonate (100 mg) were dissolved in 20 mL of deionized water. For the oil phase, 1.8 g of the Span80^®^ surfactant was incorporated into 40 mL of soybean oil. Then, the oil phase was poured over the water phase under constant stirring (Eurostar Power CV IKA, Barcelona, Spain). To initiate the internal gelation, 2.5 mL of acetic acid 4.25% *v*/*v* was added, maintaining constant stirring for 10 min, after which 2.5 mL of calcium chloride 0.45 M was added. The obtained microparticles were collected by vacuum filtration and washed with MilliQ water. 

Determination of mean diameters measured, and size distribution, were analyzed in a Mastersizer 2000 (Malvern Instruments Ltd., Malvern, UK). The mean diameter was expressed as the median value and indicated as D50. It is unnecessary and has been modified accordingly. The polydispersity index (PDI) was calculated as expressed in De Moura (2018) [31], determined using the following equation:

(1) $$PDI=\frac{d_{90}-d_{10}}{d_{50}}$$

where d_10_, d_50_, and d_90_ are the diameters at 10%, 50%, and 90% of accumulated volume, respectively.

The morphology of the obtained microparticles was analyzed using a GEMINI (FESEM) CARL ZEISS High resolution Scanning Electron Microscope (SEM) at 500×, 2000× and 10,000× magnifications.

Phenolic content present in the administered extract for encapsulation and washing water of the obtained microparticles were analyzed as described in Section 2.7., in order to establish total and superficial phenolic content, respectively. Encapsulation efficiency (EE) was determined using the following equation:

(2) $$EE\;\left(\%\right)=\frac{{PC}_{total}-{PC}_{free}}{{PC}_{total}}\times 100$$

where PC*_total_* indicates the total amount of phenolic compounds for encapsulation, and PC*_free_* indicates the non-encapsulated phenolic.

### 2.6. Formulation of a Microparticle-Enriched Yogurt

Goat (caprine) milk used for the preparation of the functional yogurt was obtained from Murciano-Granadina breed goats maintained in extensive farming conditions. Milk was divided into batches of 500 mL, replicated 3 times, and compared with milk control (100% goat milk).

Milk was preheated in the stove to 40–45 °C and maintained at 40 °C for 20 min in thermostatic bath (J.P. Selecta, Abrera, Barcelona, Spain). Probiotic yogurt cultures YO-MIX 205 LYO 250 DCU (0.024 mg) containing *Streptococcus thermophilus*, *Lactobacillus delbrueckii* subsp. *Bulgaricus*, *Lactobacillus acidophilus*, *Bifidobacterium lactis*, and microparticles rich in polyphenols (3 mg) were added and dissolved in the warm milk. Then, an incubation was carried out in sterile and enclosed bottles at 42 °C for 5 h to promote the fermentation. After the incubation, the yogurt was cooled to 4 °C. 

For extraction of phenolic compounds from the yogurt preparation, 25 g of sample was shaken for 10 min, acidified to pH 2.5 HCl 6 M, and centrifuged at 5000 rpm, 20 min, and 20 °C. Then, the obtained pellet was resuspended in 50 mL of distilled water and centrifuged under the same conditions. Both supernatants were then combined and filtered, being later mixed in duplicate with 10 mL of hexane. Samples were decanted, and the inferior phase was collected and extracted with 10 mL of ethyl acetate in triplicate. Afterward, the decantated supernatant solution was filtered with Na_2_SO_4_, evaporated in a rotary evaporator, and resuspended in 1 mL of MeOH:H_2_O (50:50, *v*/*v*). Extracted samples were filtered prior to analysis (0.22 µm nylon filter). Extraction was carried out in triplicate.

### 2.7. Characterization of Phenolic Compounds by HPLC-MS

Phenolic composition of olive leaf extracts, encapsulated formulations, and yogurt samples was assessed by HPLC-ESI-MS as previously described [32], using an Acquity UPLC System I-Class system (Waters Corporation, Milford, MA, USA), Z-Spray electrospray ionization interface (ESI, Waters Corporation, Milford, MA, USA), and a Triple Quadrupole Mass Spectrometer Xevo TQ-XS (Waters Corporation, Milford, MA, USA).

The chromatographic separation was carried out as described previously by an Acquity UPLC BEH C18™ column (2.1 × 100 mm, 1.7 µm) with a constant column oven temperature at 50 °C. The mobile phases consisted of 0.5% acetic acid in water (A) and methanol (B). The multistep linear gradient applied for the 11 min of analysis was: 5 min, 1% B; 7 min, 30% B; 7.1 min, 60% B; 9 min, 100% B; 9.1 min, 1% B, maintaining initial conditions for 2 min. The flow was 0.4 mL/min, and the injection volume was 10 µL. 

The compounds separated were monitored with a TQ-XS analyzer. The mass spectrometer was equipped with an ESI interface operating in negative ion mode, in a mass range of 72–2020 *m*/*z*. Nitrogen was used as nebulizing/ionizing and drying gas at conditions of 7 bar and 0.15 L/min, respectively. The drying temperature was set at 150/600 °C, capillary voltage of +2.20 kV, and End Plate Offset at 30 V. In order to recalibrate the mass spectra obtained during analysis to achieve a mass precision of 5 ppm, sodium formate solution was used as a calibration agent at the beginning of each analysis (*m*/*z* range of 50–1500 Da). Ion mass data were processed in the MassLynx 4.1 software (Waters Corporation, Milford, CT, USA). Identification was carried out by comparing both the retention time and MS spectral data from samples and the available commercial standards. 

Quantification of phenolic compounds contained in the extract, microparticles, and yogurt samples were also carried out by HPLC-MS analysis. Samples analyses were determined in triplicate. Stock solutions of oleuropein, rutin, hydroxytyrosol, gallic acid, diosmin, *p*-coumaric acid, ferulic acid, chlorogenic acid, and verbascoside were prepared, filtrated using 0.22 µm nylon filters and stored at −18 °C until analysis. Calibration curves were calculated by using seven points at different concentrations (10, 25, 50, 100, 250, 500 and 1000 ppb), presented in Supplementary Materials (Table S2). The quantitative content in the mentioned phenolic compounds was determined by drawing up the standard concentration as a function of the peak area. All calibration curves showed good linearity (R^2^ > 0.99).

### 2.8. Mineral Analysis

Mineral analysis was performed as previously described [33]. Samples (0.250 g for lyophilized extract samples and 1 g for liquid milk samples, microparticles, and crushed leaves) were added to 6 mL of nitric acid and placed in the digestion equipment (Multiwave 5000, Anton Paar, Austria). For removing organic matter in the samples, a temperature of 180 °C was achieved in 20 min and maintained for another 20 min. At the end of digestion, the mineralized liquid present in borosilicate tubes was collected and resuspended in 10 mL of MilliQ water. Then, mineral analysis was performed by Inductively Coupled Plasma Optical Emission Spectroscopy ICP-OES (Perkin Elmer, Optima 8300 model, Waltham, MA, USA) and by Inductively Coupled Plasma Optical Emission Mass Spectrometry ICP-OES-MS (Perkin Elmer, Inc., Waltham, MA, USA).

### 2.9. Statistical Analysis

Significant differences for phenolic content, antioxidant activity, and mineral content were evaluated using the SPSS statistical software (SPSS version 28; SPSS Inc., Chicago, IL, USA). Analysis of variance (ANOVA) and Tukey’s post hoc tests were applied to determine statistical differences (95% confidence level).

## 3. Results and Discussion

**Figure 1:**
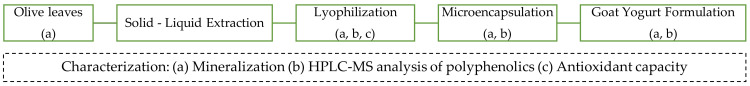
Experimental design diagram.

In this study, revalorization of an industrial by-product, namely olive leaves, was proposed through the obtention of a phenolic-rich extract and encapsulation for later implementation in the development of a functional yogurt. Experimental design is summarized in Figure 1.

### 3.1. Extraction and Analytical Characterization of Olive Leaf Phenolic Compounds

One of the main considerations for the development of new functional foods is firstly the adequacy of the process for future scalability (that is, the feasibility of the used techniques to be scalable and show a similar yield industrially) as well as its safe use for human consumption. In this sense, solid–liquid extraction is, nowadays, still an interesting industrial extraction process, requiring less specific equipment while being industrially relevant. 

In this work, conventional solid–liquid extraction was carried out on the dried and milled olive leaves at different times and sample–solvent ratios, in order to evaluate the extractive power of a specific amount of solvent as well as the evolution of phenolic compounds in the matrix. Among all the assayed conditions, the most abundant phenolic compounds reported in the literature for olive leaves, which have been related to their positive health impact, were identified and quantified, as represented in Table 1. In this sense, oleuropein, hydroxytyrosol, and verbascoside have presented great antioxidant, anti-inflammatory, immunomodulatory, and cardioprotective effects, among others [34,35,36,37]. Additionally, the monitored compounds could be related indicators of the phenolic extract stability, such as phenolic acids (coumaric, ferulic, and chlorogenic acid) and flavonoids (rutin and diosmetin).

When observing the global phenolic extraction, no significant differences were observed at increasing amounts of sample while maintaining the extraction volume for most of the phenolic compounds. This could indicate a limiting role of the extraction solvent in the increased extractability of the desired compounds. 

Nevertheless, extraction resulted in the obtention of high quantities of the compounds of interest when not stored over periods of time. Overall, oleuropein seems to be the most abundant compound, comprising 95% of the total identified content, followed by verbascoside (4%) and hydroxytyrosol (0.6%). Oleuropein, which was present in 97.07–122.77 mg/g dried leaf, seems to be high when compared with the previous literature on solid–liquid conventional extraction with solvents with different methanol content, which have ranged from 0.54 mg (80% methanol, [38]) to 14.4 mg/g dried leaf (50% methanol, [39]) and 72.08 mg/g dried leaf assisted with Microwave-Assisted Extraction (MAE) (80% methanol, [40]). Additionally, it seems to be in line with that obtained in macerations with 80% ethanol, reaching up to 109.6 mg/g dried leaf [41,42]. 

As for verbascoside, obtained in 4.38–5.32 mg/g dried leaf, it seems to be similar with that reported for macerations with 80% ethanol, which obtained a range of 2.08–2.32 mg/g dried leaf [41]. Finally, hydroxytyrosol content (0.66–0.77 mg/g dried leaf) appears to be slightly higher than similar extractions in the literature, which have ranged from 0.14 (methanol) to 0.43 (80% methanol) mg/g dried leaf [10,40]. In this sense, the extraction procedure has proved to allow for an adequate obtention of phenolic compounds.

Additionally, the impact of olive leaf storage on the stability of phenolic compounds and, thus, its extractability, was also assessed in extractions carried out over a 2-month period. In this case, independently of the selected extraction amounts, a significant, although not intense, degradation of phenolic compounds can be observed over time. Specifically, this degradation seems to be more prominent during the first month of storage of the leaves and is mainly focused on the degradation of oleuropein (reduced in 1% throughout a 2-month period), while it becomes less intense in the third extraction. 

Stability of phenolic compounds has shown to be dependent on environmental conditions during processing and storage, which include pH and temperature. Indeed, a degradation of compounds as a result of their evolution under environmental conditions is to be expected and has been previously reported for the storage of phenolic olive leaf extracts [41]. This may be related to the exposure of phenolic compounds to environmental conditions and to the impact of the drying process on their resulting stability. Additionally, the presence of enzymes found in the olive structures may have an effect on the observed results. In this sense, some phenolic degrading enzymes could be compartmentalized in the leaf, as was observed for olive fruit cell [43]. However, drying and storage at the assayed conditions may enhance the rupture of cell structures, allowing for the interaction with studied polyphenols. Thus, even with a reduced activity, enzymes present in this plant’s structures could also be considered, as the polyphenol oxidase. In this sense, although dehydrated, phenolic content could further evolve due to environmental storage conditions.

### 3.2. Antioxidant Activity

Evaluation of the antioxidant activity of the obtained extracts by different antioxidant methods, namely DPPH, ABTS, and TEAC, was also carried out. The results for the extracts obtained at different storage times are presented in Table 2.

Antioxidant activity results of the extracts agree with the above-described phenolic extraction yield. Antioxidant activity was high for all the evaluated assays, with the best results being observed for FRAP. The obtained data for the first extraction are similar to or higher than those observed in the previous literature for ethanolic extracts, where FRAP results have ranged from 75.7 ± 0.9 to 109.5 ± 5 mg TE/g dry weight and 4.5 ± 0.4 to 8.3 ± 0.5 mg TE/g dry weight for ABTS [42]. Additionally, these results seem to be in line with Hayes et al., 2011, where antioxidant capacity of a commercial olive leaf extract reported 379.3 mg TE/g DW for ABTS and 300.1 mg TE/g DW for FRAP [44]. Additionally, DPPH inhibition percentages have been found to be around 90% for several alcoholic extracts of olive leaves, in line with the 86.4% presented in this study [45,46]. This emphasizes the potential of this simple yet scalable extraction process for the obtention of bioactive compounds of interest from olive by-products.

In this sense, antioxidant capacity was the highest for extraction of lower amounts of olive leaves, remarking the importance of the sample–solvent proportions in the observed bioactivity.

Indeed, for the DPPH assay, the highest antioxidant activity was found for the first extractions at different proportions, as no significant differences were observed in this case. Considering 2 g/100 mL, storage did not present a significant effect on this value, while a reduction was observed for 5 g/100 mL at 1- and 2-month storage and for 12.5 g/100 mL at 1-month storage. However, when comparing the behavior of these proportions at different storage times, 2 g/100 mL seems to consistently maintain the highest antioxidant activity, the difference being more significant at 12.5 g/100 mL.

On the other hand, the FRAP assay has led to different observations, in which although for 2 g/100 mL antioxidant activity seems to be reduced, for the other proportions, extraction at storage time of 2 months (S8 and S9) showed the highest activity among the evaluated samples. 

Finally, for the ABTS assay for 5 g/100 mL and 12.5 g/100 mL proportions, antioxidant activity seems to decrease at one month, only to slightly increase in the second month. However, among the initial extraction conditions, 2 g/100 mL reported the best antioxidant results. 

Overall, antioxidant activity seems to be reduced as storage time is increased, with the exception of FRAP results. As the nature of the evaluated assays is different and depends on the specific action of the presented compounds, differences among assays are to be expected. Indeed, the observed activity of each compound appears to be related to its specific structure. In this sense, structure–antioxidant activity of phenolic compounds has been linked with the number and location of hydroxyl groups in the phenolic ring, with increasing its potential when presented in ortho- or para-positions (catechol) as related to a higher electron-donating ability [47,48]. The presence of hydroxyl groups in this configuration has been observed in all the phenolic compounds identified in the extract, being a determinant factor for the observed results. Specifically, presence in the ortho-position can be found in hydroxytyrosol, chlorogenic acid, oleuropein, rutin, and verbascoside, the latter exhibiting two catechol groups. Additionally, other functional structures such as C=O (oxo) or OAc have also demonstrated an association with high antioxidant potential, also found in some of the mentioned phenolics. 

As the considered extraction technique and sample are considered, these data appear to be related to their specific concentration and the modification in phenolic content in the storage olive leaves, favoring the degradation of the identified compounds into other molecules that differentially contribute to the observed antioxidant results. Indeed, each specific compound presents a distinct antioxidant capacity for different assays, as previously reported, and their degradation may be responsible for the observed decrease at 1-month storage, but with the obtention at longer storage periods of compounds that may contribute to a higher electron-transference antioxidant activity, increasing the observed FRAP values. As both the DPPH and ABTS assays are based on a different molecular process, these degradation products could not have contributed in the same manner as in this case. However, the synergistic effect of the specific phenolic composition may also be a determinant factor on the observed results. These data further emphasize the importance of understanding the molecular mechanisms underlying the antioxidant properties of phenolic compounds and their specific interactions in the observed bioactivity.

Additionally, environmental and technological aspects should also be taken into consideration, including the influence of the drying process in the stability of the phenolics and the effect of the storage temperature on the presence of enzymatic activity of enzymes such as polyphenol oxidase and peroxidase, whose activity has been shown to be significantly reduced at lower temperatures [49]. Furthermore, storage temperature does not eliminate the potential degradation, as storage of lyophilized olive phenolics has shown significant antioxidant activity reductions when submitted to long storage periods, superior to 21 days, even at lower (4 °C) storage temperatures [41].

### 3.3. Microencapsulation of Olive Leaf Extracts by Ionic Gelation

As for the previously reported results for phenolic content and antioxidant capacity of the evaluated extracts at different conditions, positive data across the different assays showed S1 as the optimal extract for the microcapsules production and development of the posterior functionalized dairy product. The incorporation of the phenolic-rich extract into an encapsulated formulation is expected to improve the stability and structural integrity of the bioactive compounds throughout the industrial processes and gastrointestinal environment. 

**Figure 2:**
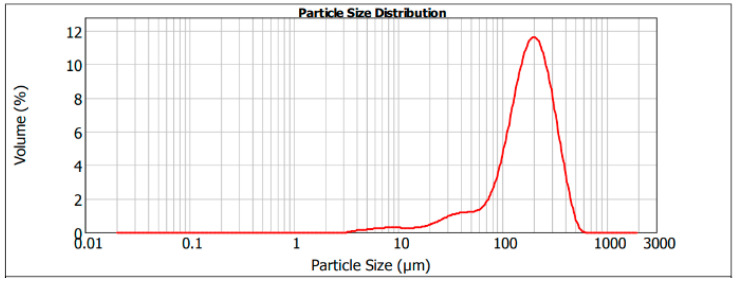
Particle size distribution of the olive leaf extract microcapsules.

Concerning the physical characterization of microparticles, the results of size distribution of the particles generated by encapsulation of the phenolic compounds are presented in Figure 2. Mean diameter D [4,3] resulted in 191.48 µm, while the median (D50) was 180.91 µm. The polydispersity index (PDI) was 1.48.

The experimental conditions on the emulsion production, including maintenance of concentration of surfactant and stirring, allowed for obtaining microparticles with a size range under 330 µm. This emulsion produced particles that exhibited a multimodal size distribution, in concordance with the previous literature [50,51]. This variation, although parameters in the procedure were maintained constant, may be explained through the development of an irregular gelation process, a consequence of a differential release of calcium ions [52]. However, this effect does not seem to be of high significance, as the PDI appears to be low, with most of the volume found between 100 and 300 µm.

This encapsulation procedure appears to provide a similar size range to that reported in the previous literature for ionic gelation. The presented results fall among the presented D50 values (400 μm) in Yamdech et al., 2012, for mulberry fruit extract encapsulation, although slightly lower [53]. Additionally, chitosan encapsulation also presented microparticles in the range of those presented in this study (160.58–206.52 μm) [54]. Overall, the obtained size distribution appears to be inferior to other studies, where the size was up to the range of millimeters [50,55,56].

**Figure 3:**
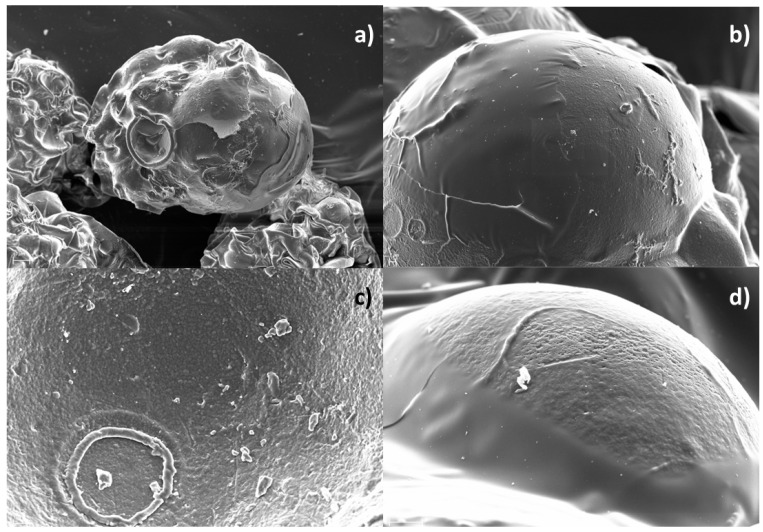
SEM microphotograph of olive leaf extract alginate microcapsules at different magnifications: (**a**) 500×, (**b**) 2000×, (**c**) 5000×, (**d**) 10,000×.

With respect to morphology, the ionotropic gelation process resulted in a successful production of phenolic-loaded microparticles. The obtained beads presented a spherical shape, with few superficial irregularities and porousness, resulting in a high smoothness (Figure 3). The presence of irregularities on this desired morphology has also been observed in Flamminii et al., 2021, for alginate-based ionic gelation microparticles of olive leaves extract, and Belscak-Cvitanovic et al., 2016, for dandelion phenolic extract encapsulated with alginate and pectin, as varying CaCl_2_ percentages and different encapsulation agents seem to influence these structural aspects [52,54]. Furthermore, the drying process resulted in a higher tendency for aggregation between structures. Indeed, this process has presented a significant effect in the morphology alteration of alginate-based microparticles, which has previously resulted in collapsed structures, irregular shapes, and heterogeneous surfaces [51,57,58]. Nevertheless, in our study, the obtained capsules were able to maintain structural integrity throughout the drying process, thus exhibiting their resistance to these taxing processes.

### 3.4. Encapsulation Efficiency of the Phenolic-Rich Olive Leaf Extract

As depicted in previous sections, the most optimal extraction conditions were those assayed for sample S1, with the most reduced storage time and concentration of olive leaf and, therefore, were the ones considered for the encapsulation process. In this sense, total phenolic content in the extract, encapsulated amount, and efficiency (%) are presented in Table 3.

Previous morphological characterization results have demonstrated the potential of the selected encapsulation process in the obtention of microparticles with a desirable morphology and size. However, its ability to incorporate the desired compound into a protective structure also needs to be considered. Thus, the encapsulation efficiency has been used as an insightful parameter in the evaluation of its potential and adequacy for the used extract, and it is presented in Table 3. In this sense, we have focused the analysis on the most abundant compounds detected in this extract, which have been related to its positive health connotation.

Overall, although it may differ depending on the specific compound, encapsulation efficiency presents remarkable values, above 90% for all cases, showing its adequacy for the selected phenolic extract. Notably, for hydroxycinammic acids, such as *p*-coumaric acid and ferulic acid, all of its content has been incorporated into the encapsulated formulations, thus rendering the process highly effective for these compounds, with only a slight reduction for chlorogenic acid (97.60%). This positive effect has been previously observed for ionotropic gelation with alginate in combination with whey protein as a co-structural agent, reaching up to 89% efficiency [52].

Additionally, oleuropein, hydroxytyrosol, and verbascoside, the most abundant bioactive compounds in the presented extract, also show an outstanding encapsulation efficiency. This contrasts with Flamminii et al., 2021, where although hydroxytyrosol was encapsulated in a 80%, this efficiency was lower for both oleuropein and verbascoside in alginate microparticles, and the presented results are more similar with what was obtained for co-encapsulation with pectin [57].

These values are increased when compared to the variety observed in the literature for other phenolic extracts, where total phenolic encapsulation has ranged from 41% to 85%. Indeed, in some cases the observed efficiency is similar to that obtained with co-encapsulation of alginate with other wall materials, which is quite promising.

In this scenario, the nature of the matrix material has proved to be an essential factor in ensuring bioactive compounds entrapment and stability and, therefore, may have an effect on the variations of encapsulating efficiency, between studies. Alginate has proved to be a successful encapsulation agent for the incorporation of phenolic compounds from diverse sources using ionotropic gelation. However, efficiency of alginate for encapsulation of phenolic compounds from different sources may exhibit some variations depending on their structure. This has been related to the interactions established between the molecules, such as hydrogen bonds. In this sense, the presence of hydroxyl groups has positively correlated with a higher formation of hydrogen bonds with polysaccharides [59]. Abundance of the mentioned functional group in the identified molecules may explain the high efficiency of the presented process. Indeed, due to this aspect, verbascoside has previously shown increased values of encapsulation efficiency with alginate compared to other olive leaf phenolic compounds, such as oleuropein [54,60].

Additionally, the ability of the encapsulating material to produce smooth structures may also present an effect on the efficiency of the process. Porous structures, originated from the specific interactions of the selected protective material, may lead to a loss of bioactive molecules and, therefore, a reduction in the observed encapsulation efficiency [61]. Thus, the smooth surface of these microparticles may contribute to its overall effectiveness.

When considering encapsulation efficiency of oleuropein (the major phenolic compound present in olive leaf extracts) by different methodologies, the presented encapsulation efficiencies seem to be quite promising. Indeed, the highest encapsulation efficiency for oleuropein when using freeze-drying as an encapsulating method was 99.23 ± 0.16%, quite similar to that proposed in our study [61]. In addition, encapsulation efficiency for oleuropein was 60.8% in olive leaf microcapsules encapsulated by spray-drying with alginate [62].

Thus, the presented results support the potential of the encapsulation process for the selected olive leaf extract. The encapsulation effectiveness of the capsules was established by the matrix material’s ability to retain bioactive compounds and confirms process success.

### 3.5. Functional-Goat Yogurt Formulation by the Incorporation of Microencapsulated Olive-Leaf Phenolic Extract

The applicability of the obtained microcapsules to food products was evaluated through the incorporation of these formulations into a functionalized goat yogurt, to test their stability in the production process. Thus, the main phenolic compounds found in the phenolic extract were quantified for the functional yogurt and compared to a control sample, as presented in Table 4. The presence in the control yogurt of several phenolics could be due to the specific type of farming to which the goats were subjected, and thus directly derived from their specific diet and not synthesized by the mammal. Indeed, the abundance of olive plantations in the area could be related to their increase in oleuropein, a secoiridoid mainly presented in different structures derived from the olive tree, as they could have been ingested by the mammals [63]. As for the functional yogurt, among the selected phenolics, only hydroxytyrosol and oleuropein were present in the functional yogurt, at low quantities. The presence of both compounds, being the most abundant in the olive leaf extract, could be related to their observed encapsulation efficiency, as this presence is not observed for the control yogurt sample for hydroxytyrosol. This could lead to a superficial presence of these compounds that may diffuse into the yogurt media. Additionally, for oleuropein, the observed presence is a relation of the natural presence of this compound in the yogurt and the encapsulation efficiency.

Overall, the presented results show the stability of the encapsulated formulation of the extract under the acidic environment of the fermented product, as well as its ability to endure the technological production process, rendering the obtained microcapsules as optimal for the selected product [64]. Additionally, encapsulation of these compounds is desirable in a food matrix as presented, which contains a high presence of lactic acid bacteria. Phenolic compounds have proved to be highly prebiotic molecules, as well as presenting other bioactive properties for which they may have gained public interest and can be easily metabolized by bacteria. As the microcapsules are incorporated simultaneously into the starter cultures in the obtention of the yogurt, were these compounds to be accessible and presented freely in the food matrix, they would have been fermented, thus not accessible in the final product. The encapsulation process has also efficiently protected these compounds from bacteria-mediated degradation, ensuring the functionalization of the product. Thus, these results highlight the protective effect that the obtained formulations may present on the selected phenolic extract, allowing for their preservation and controlled delivery, which may increase its bioaccessibility.

Indeed, sodium alginate has proved to be a desirable encapsulation agent that not only is safe for consumption, it also has proved a preserving role in phenolic compounds during several industrial processes. However, ionic gelation encapsulation seems to be underexplored for the selected plant matrix, as reflected by the lack of literature regarding this process on olive leaf extracts with application in dairy products. Nevertheless, the previous literature has exhibited the protective ability and stability in the encapsulation of *Ceratonia siliqua* L. hydroethanolic extract, where the encapsulated formulation efficiently protected the bioactive compounds after their incorporation into a functional yogurt formulation [65]. Similar results have also been presented for the same product for the encapsulation of *Rubus ulmifolius* flower extracts [66]. Nonetheless, in both studies, the microparticles were introduced into already obtained yogurt. In the present study, furthering this research, we have determined the main phenolic compounds found in olive leaf extracts, secoiridoids, phenolic alcohols, and phenylethanoids/phenylpropanoids throughout the functional yogurt production process with olive leaf microcapsules. In this sense, these results have shown that this stability is also achieved when introducing these microparticles during the processing, which indicates the scalability potential of these formulations.

Furthermore, the possibilities are not only limited to the production process, as this stabilization could be also projected to storage time, as has been observed for encapsulated aromatic plant extracts [61]. Furthermore, these formulations have also increased the stability of phenolic compounds on their incorporation into several types of fortified cheese [8,67].

Our study has shown the potential of olive leaf extracts, not only as sources of bioactive compounds with outstanding applications but the adequacy of the encapsulation process for ensuring stability of these compounds in the development of a functionalized yogurt. This could lay the foundation for furthering the research on the revalorization of this industrial by-product into functionalized dairy fermented products.

### 3.6. Mineral Content in the Analyzed Samples

In addition to the content in bioactive phytochemicals, the effect of the inclusion of the obtained microparticles into the micronutrient content of the functional yogurt was evaluated. Concentrations of mineral elements in the olive leaves, extracts, microparticles, and enriched yogurts, classified into macroelements and microelements, are presented in Table 5. As the presented minerals are essential for the correct functioning of the biological responses and activity of enzymes, analysis of the potential of the studied by-product as a mineral source is studied.

Among the evaluated macroelements, the most abundant for all considered samples were Ca, K, Mg, and S, followed by P and Fe. Indeed, calcium appears to be the predominant mineral in olive leaves 9.409 ± 0.045 mg/g, in accordance with the data reported by Lee et al., 2005 (9.296 mg/g dried leaves) and Bahloul et al., 2014 (9.25 mg/g dried leaves) [68,69]. The presence of this compound in dietary supplements may have a use for the prevention of calcium deficiencies, improving bone health. On the other hand, although Fe is not the most abundant element, its presence in the dried olive leaves must be considered. In fact, consumption of 1 g of olive leaves leads to the obtention of 2% of the Population Reference Intake (PRI) and 100% when consuming 50 g, in line with the results presented in Cavalheiro et al., 2015 [70].

As for the microelements, the most abundant were Cu, with a similar content to that previously observed for olive leaves, and Mn [70]. Additionally, Pb and U are present in trace amounts. This may be an indication of the presence of contamination on the soil, as Pb accumulation has been related to anthropogenic origins, and its affinity with organic and colloidal materials improves their uptake by plants [71]. However, its presence is still in rather low quantities when compared to what has been observed in the literature for spice crops, such as rosemary, where Pb has been present in concentrations up to 9.38 mg/kg [71]. Also, content in U in the present study is lower than observed for several foods [72]. Therefore, the observed concentration in our study should not raise any health concerns with its consumption. The no-observed-adverse-effect levels (NOAEL) for uranium depend on the specific form in which it is presented and specific alterations to be observed, with one of the lowest NOAEL concentrations being observed at 40 mg U/kg/day for uranium peroxide, while this value for Pb is 57 µg Pb/kg/day [72,73]. In this sense, the observed concentrations are below these levels, reinforcing the security in its consumption.

On the other hand, concentration of the different quantified compounds tends to vary in the extract. This differential extraction of minerals could be related to the nature and affinity with the extraction solvent. Indeed, differences were observed depending on the extraction solvent when comparing the distribution of minerals in moringa extracts [74].

Furthermore, different sample–solvent proportions were evaluated. This parameter does not seem to exhibit a significant influence on the presence of some macro- and micro-minerals, as is the case for Fe, P, S, Mn, Cu, Rb, and U, coinciding with that stated above for extractability of phenolic compounds. While significant differences have been observed between different ratios for some compounds, their content seems to mainly decrease as sample content increases (as is the case for B, Zn, and Se). Specifically, for micromineral differences, when observed, they resulted in a reduction in content with a higher presence of olive leaves in the extraction. Thus, the lowest concentration of olive leaves seems to have a more desirable behavior, in line with that mentioned in previous sections.

Additionally, the content of all major minerals in the microparticles is significantly different from that observed in the different extracts. Their inclusion also seems to be dependent on the specific molecule, both Na and Ca being the best encapsulated, while B is not as present as it is in the extracts.

As for the functionalized yogurt, inclusion of the microparticles resulted in an increased content for most of the considered macrominerals, while microminerals tended to remain similar to the control, with the exception of Fe and P, where presence in the yogurt is slightly lower. This appears to be associated to the observed content in both the extract and encapsulated formulations and the possible effect of its incorporation. In this sense, increased presence of the former minerals in the microparticles allows for a significant effect on the yogurt composition even when administered at low quantities, such as is the present case. However, microminerals are presented in trace amounts, not significant to efficiently affect the nutritional profile of the functionalized yogurt. Thus, and although in small increments, introduction of the microparticles into the proposed functional food does not only allow for the inclusion of bioactive compounds that may have a positive impact on health but also positively influences its mineral profile.

Overall, the results derived from this preliminary study have pointed out the interesting potential of the selected extract and the microencapsulated formulation in the incorporation and development of a functional product which may have potential beneficial health effects. Future studies are warranted to assess the effect of the production process scale-up on bioactive phenolics and to establish the behavior of the goat yogurt formulation under gastrointestinal conditions both in vitro and in vivo, as well as to evaluate the bioactive potential of its consumption.

## 4. Conclusions

In this work, the influence of different sample–solvent ratios and storage times was evaluated on the green extraction of phenolic compounds from olive leaves as industrial by-products of great potential, for their later encapsulation and incorporation into a functionalized yogurt formulation. In this sense, the best extraction conditions included low sample proportions and a reduced storage time, as a result of solvent saturation and degradation of the bioactive compounds when maintained at storage conditions in their original plant structures. The encapsulation process allowed for a high incorporation of the main phenolic compounds that translated into their protection when submitted to the fermentation process. Thus, the presented microencapsulated extract shows great potential for its incorporation into early industrial stages of the obtention of fermented dairy products for the production of functional foods of great health potential.

## Figures and Tables

**Table 1:** Polyphenolic content, expressed in mg/g dried olive leaf using different sample–solvent proportions.

Bioactive Compounds	S1	S2	S3	S4	S5	S6	S7	S8	S9
Hydroxytyrosol	0.72 ± 0.05 ^ab^	0.77 ± 0.02 ^a^	0.67 ± 0.05 ^ab^	0.61 ± 0.07 ^b^	0.49 ± 0.04 ^c^	0.58 ± 0.06 ^bc^	0.5 ± 0.05 ^c^	0.44 ± 0.01 ^c^	0.48 ± 0.07 ^c^
P-coumaric acid	0.028 ± 0.003 ^a^	0.032 ± 0.002 ^a^	0.029 ± 0.002 ^ad^	0.03 ± 0.01 ^ab^	0.027 ± 0.001 ^bc^	0.026 ± 0.001 ^bd^	0.0218 ± 0.0005 ^a^	0.03 ± 0.001 ^c^	0.036 ± 0.002 ^e^
Ferulic acid	0.011 ± 0.001 ^a^	0.01 ± 0.002 ^a^	0.0066 ± 0.0002 ^b^	0.006 ± 0.001 ^b^	0.0067 ± 0.0005 ^b^	0.0062 ± 0.0002 ^b^	0.009 ± 0.001 ^ac^	0.007 ± 0.002 ^bc^	0.0075 ± 0.0005 ^b^
Chlorogenic acid	0.09 ± 0.01 ^ab^	0.102 ± 0.003 ^a^	0.06 ± 0.01 ^c^	0.08 ± 0.01 ^b^	0.06 ± 0.01 ^c^	0.08 ± 0.01 ^b^	0.031 ± 0.004 ^d^	0.047 ± 0.003 ^cd^	0.05 ± 0.01 ^c^
Oleuropein	113 ± 5 ^a^	123 ± 6 ^a^	97 ± 3 ^b^	81 ± 7 ^c^	81 ± 7 ^c^	85 ± 10 ^c^	62 ± 6 ^d^	69 ± 1 ^cd^	88 ± 13 ^bc^
Diosmin	0.09 ± 0.01 ^ab^	0.11 ± 0.01 ^a^	0.102 ± 0.004 ^ab^	0.07 ± 0.01 ^c^	0.08 ± 0.01 ^c^	0.09 ± 0.01 ^b^	0.06 ± 0.01 ^c^	0.063 ± 0.003 ^c^	0.09 ± 0.02 ^ab^
Rutin	0.56 ± 0.03 ^ab^	0.69 ± 0.03 ^c^	0.57 ± 0.03 ^a^	0.44 ± 0.05 ^bc^	0.45 ± 0.04 ^abc^	0.52 ± 0.07 ^ab^	0.38 ± 0.05 ^c^	0.4 ± 0.01 ^c^	0.5 ± 0.08 ^abc^
Verbascoside	4.4 ± 0.3 ^ab^	5.3 ± 0.2 ^c^	4.5 ± 0.2 ^ab^	3.8 ± 0.5 ^bd^	3.7 ± 0.3 ^bd^	4.4 ± 0.5 ^ab^	2.9 ± 0.3 ^d^	3.37 ± 0.09 ^d^	5.2 ± 0.3 ^a^

S1: 2 g/100 mL, no storage time; S2: 5 g/100 mL, no storage time; S3: 12.5 g/100 mL, no storage time; S4: 2 g/100 mL, 1 month storage; S5: 5 g/100 mL, 1 month storage; S6: 12.5 g/100 mL, 1 month storage; S7: 2 g/100 mL, 2 months storage; S8: 5 g/100 mL, 2 months storage; S9: 12.5 g/100 mL, 2 months storage. Different letters among the same row indicate the presence of statistically significant differences.

**Table 2:** In vitro antioxidant activity of extracts obtained at different times and sample–solvent ratios determined by DPPH, FRAP, and TEAC, expressed as µmol of Trolox Equivalent (TE) per g of dried olive leaf.

	S1	S2	S3	S4	S5	S6	S7	S8	S9
DPPH	632 ± 54 ^a^	608 ± 12 ^a^	474 ± 6 ^bc^	554 ± 26 ^ad^	451 ± 15 ^bc^	405 ± 32 ^b^	557 ± 14 ^ad^	505 ± 41 ^cd^	432 ± 7 ^bc^
FRAP	858 ± 19 ^a^	834 ± 5 ^ab^	822 ± 56 ^ab^	623 ± 7 ^c^	628 ± 1 ^c^	836 ± 92 ^ab^	776 ± 46 ^b^	723 ± 6 ^b^	1040 ± 4 ^d^
TEAC	448 ± 4 ^a^	401 ± 7 ^b^	407 ± 1 ^b^	414 ± 7 ^b^	382 ± 5 ^c^	486 ± 17 ^d^	408 ± 22 ^b^	369 ± 2 ^c^	395 ± 6 ^bc^

S1: 2 g/100 mL, no storage time; S2: 5 g/100 mL, no storage time; S3: 12.5 g/100 mL, no storage time; S4: 2 g/100 mL, 1 month storage; S5: 5 g/100 mL, 1 month storage; S6: 12.5 g/100 mL, 1 month storage; S7: 2 g/100 mL, 2 months storage; S8: 5 g/100 mL, 2 months storage; S9: 12.5 g/100 mL, 2 months storage. Different letters among the same row indicate the presence of statistically significant differences.

**Table 3:** Extract composition, encapsulated phenolic content, and encapsulation efficiency in the obtained microcapsules.

Bioactive Compounds	Extract Phenolic Content (mg/g of Extract)	Encapsulated Content (mg/g Microparticles)	Encapsulation Efficiency (%)
Hydroxytyrosol	2.2 ± 0.2	2.05	91.72
P-coumaric acid	0.09 ± 0.01	0.09	100.00
Ferulic acid	0.04 ± 0.002	0.04	100.00
Chlorogenic acid	0.28 ± 0.02	0.27	97.60
Oleuropein	353 ± 15	339.22	96.10
Diosmin	0.29 ± 0.02	0.28	96.26
Rutin	1.7 ± 0.1	1.69	96.81
Verbascoside	13.7 ± 0.9	13.25	97.01

**Table 4:** Phenolic content of the obtained enriched goat yogurt expressed as µg of polyphenols per 100 g of yogurt.

Bioactive Compounds	Control Yogurt	Functional Yogurt
Hydroxytyrosol	nd	0.062 ± 0.004
P-coumaric acid	nd	nd
Ferulic acid	nd	nd
Chlorogenic acid	0.774 ± 0.007	nd
Oleuropein	0.69 ± 0.03	2.75 ± 0.192
Diosmin	nd	nd
Rutin	1.24 ± 0.04	nd
Verbascoside	nd	nd

nd: non detected.

**Table 5:** Mineral content presented in the dried leaves, selected extract, and microparticles, as well as the functionalized and control yogurts.

	Minerals	Dried Olive Leaves (µg/g)	Extract (µg/g)	Microparticles (µg/g)	Control Yogurt (mg/100g)	Functional Yogurt (mg/100 g)
Macrominerals	B	10.7 ± 0.2 ^a^	119 ± 1 ^b^	3.293 ± 0.002 ^c^	0.175 ± 0.001 ^d^	2.01 ± 0.08 ^e^
	Ca	12792 ± 231 ^a^	3838 ± 69 ^b^	6963 ± 37 ^c^	80.1 ± 1.2 ^d^	82.9 ± 1.2 ^e^
	Fe	291 ± 9 ^a^	13.7 ± 0.9 ^b^	9.6 ± 0.1 ^b^	0.098 ± 0.001 ^d^	0.073 ± 0.003 ^e^
	K	4767 ± 139 ^a^	10459 ± 109 ^b^	525 ± 1 ^c^	122.9 ± 0.6 ^d^	132.6 ± 1.8 ^e^
	Mg	1224 ± 47 ^a^	2625 ± 40 ^b^	21.2 ± 0.1 ^c^	11.491 ± 0.197 ^d^	12.1 ± 0.3 ^e^
	Na	69 ± 6 ^a^	311 ± 4 ^b^	3090 ± 2 ^c^	61.3 ± 0.2 ^d^	66.3 ± 2.7 ^e^
	P	611 ± 12 ^a^	572 ± 17 ^a^	25.1 ± 0.1 ^b^	67.3 ± 0.6 ^d^	64.4 ± 1.9 ^e^
	S	1265 ± 39 ^a^	1076 ± 34 ^b^	64.3 ± 0.01 ^c^	25.0 ± 0.1 ^d^	25.5 ± 0.8 ^d^
Microminerals	Mn	32.935 ± 9.4 ^a^	56.5725 ± 4.5 ^b^	0.35 ± 0.04 ^c^	0.0031 ± 0.0003 ^d^	0.0039 ± 0.0011 ^d^
	Cu	38 ± 7 ^a^	41 ± 2 ^a^	0.308 ± 0.027 ^b^	0.0038 ± 0.001 ^d^	0.0044 ± 0.0005 ^d^
	Zn	8 ± 4 ^a^	33 ± 8 ^b^	0.84 ± 0.07 ^a^	0.33 ± 0.04 ^d^	0.41 ± 0.02 ^d^
	Se	0.12 ± 0.02 ^a^	0.13 ± 0.01 ^a^	0.013 ± 0.002 ^c^	0.0037 ± 0.0004 ^d^	0.0036 ± 0.0008 ^d^
	Rb	1.9 ± 0.6 ^a^	4 ± 0.1 ^b^	0.04 ± 0.002 ^c^	0.15 ± 0.01 ^d^	0.17 ± 0.01 ^d^
	Pb	0.42 ± 0.1 ^a^	0.34 ± 0.08 ^a^	0.11 ± 0.01 ^b^	0.0004 ± 0.0001 ^d^	0.0008 ± 0.0005 ^d^
	U	0.012 ± 0.002 ^a^	0.0021 ± 0.0001 ^b^	0.0138 ± 0.0003 ^a^	0.000031 ± 0.000003 ^d^	0.000045 ± 0.00001 ^d^

Different letters among the same row indicate the presence of statistically significant differences between data.

## Data Availability

All the data generated by this research have been included in the article. For any assistance, it is possible to contact the corresponding authors.

## Supplementary Materials

The following supporting information can be downloaded at: https://www.mdpi.com/article/10.3390/foods13071036/s1, Table S1: FRAP, DPPH, and ABTS calibration curves; Table S2: Standards and calibration curves used in the quantification of phenolic compounds.
