# Supplementary material for: Exploring the Nutritional and Bioactive Potential of Olive Leaf Residues: A Focus on Minerals and Polyphenols in the Context of Spain’s Olive Oil Production
Source: Foods. 2024 Mar 28;13(7):1036. doi: 10.3390/foods13071036 (PMC11012209; doi:10.3390/foods13071036)
Supplement: Supplementary file 1 [file foods-13-01036-s001.zip › foods-2920802-supplementary.pdf]

Table S1. FRAP, DPPH, and ABTS calibration curves.

**Antioxidant Assay**	**Calibration curve**
FRAP	y = 6,1115x - 0,0235; R^2 = 0,9998
DPPH	y = 146,71x + 2,5309; R^2 = 0,9974
ABTS	y = 237,19x + 1,0381; R^2 = 0,9975

Table S2. Standards and calibration curves used in the quantification of phenolic compounds.

**Standard**	**Calibration curve**
Hydroxytyrosol	y = 80.2918x – 21.5442; R^2 = 0.9980
p-Coumaric acid	y = 97.2604x + 194.613; R^2 = 0.9992
Ferulic acid	y = 106.1510x + 136.121; R^2 = 0.9990
Chlorogenic acid	y = 107.0420x + 136.583; R^2 = 0.9993
Oleuropein	y = 126.4420x + 129.414; R^2 = 0.9998
Diosmin	y = 690.21x – 265.771; R^2 = 0.9997
Rutin	y = 207.4900x + 324.215; R^2 = 0.9990
Verbascoside	y = 193.265x + 558.578; R^2 = 0.9990
